# 884. The Role of a Novel Inpatient Consultation Service for Persons Experiencing Homelessness in Detroit, Michigan: Finding Ways to Close the Gap

**DOI:** 10.1093/ofid/ofad500.929

**Published:** 2023-11-27

**Authors:** Seema Joshi, Nedda Elewa, Andrew J Failla, Madison Mervis, Katerina Furman, Sanjna Ghanshani, Nyla N Leonardi, Roxanne Ilagan, Marcus Zervos, Richard Bryce

**Affiliations:** Henry Ford Hospital, Detroit, Michigan; Wayne State University School of Medicine, Detroit, Michigan; Henry Ford Hospital, Detroit, Michigan; Wayne State University School of Medicine, Detroit, Michigan; Wayne State University School of Medicine, Detroit, Michigan; Wayne State University School of Medicine, Detroit, Michigan; Wayne State University School of Medicine, Detroit, Michigan; Wayne State University School of Medicine, Detroit, Michigan; Henry Ford Hospital, Detroit, Michigan; Henry Ford Hospital, Detroit, Michigan

## Abstract

**Background:**

People experiencing homelessness (PEH) face inequitable social determinants of health that impact primary care prevention, complications related to infectious diseases (ID), and hospitalizations. The purpose of Street Medicine Organizations is to provide accessible medical care to PEH where they reside. We describe a uniquely developed hospital consult service (HCS) targeted for PEH.

**Methods:**

This HCS took place in an 877-bed quaternary care hospital in Detroit, MI and occurred from 07/2022 – 03/2023. Inclusion criteria were PEH (defined as living unsheltered, sheltered, or with unstable housing). **Figure 1a** describes HCS timeline and objectives and aims of the HCS are shown in **Figure 1b**. The HCS template is shown in **Figure 2.** Healthcare screening (HS) recommendations were provided to the primary team, and discharge support including harm reduction education and community resources were provided to the patient. The outcomes evaluated included HS and vaccine orders and follow-up success on street medicine runs.

**Figure:**
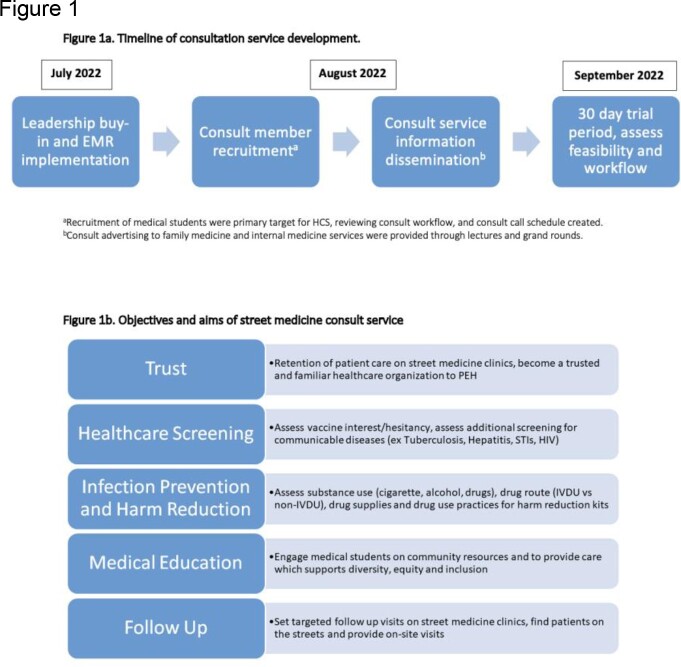

**Figure:**
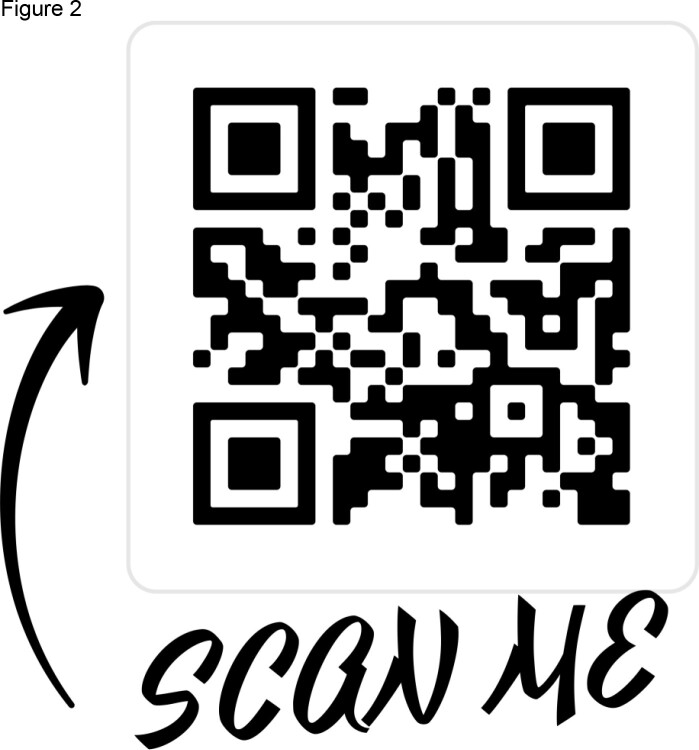

Consult note template

**Results:**

60 patients were evaluated with the HCS from 09/2022 – 03/2023. Of those, 43 consults were completed prior to patient discharge. Patient characteristics are shown in **Table 1**. Median population age was 50 years, 44 (73.3%) were men and 46 (76.7%) were Black. Majority of patients seen (48.8%) were unsheltered. 31 patients (76.7%) had last seen a primary care provider > 12 months ago. Patient were more inclined to receive the influenza (30.2%) and pneumococcal (30.2%) vaccine compared to the COVID-19 vaccine (7%). 25 patients (58.1%) admitted to polysubstance use. There were recommendations placed for 23 (53.4%) of patients in which 11 (47.8%) were executed successfully prior to discharge. 17 (39.5%) patients had a scheduled follow up visit at a specific location and time on the street.

**Figure:**
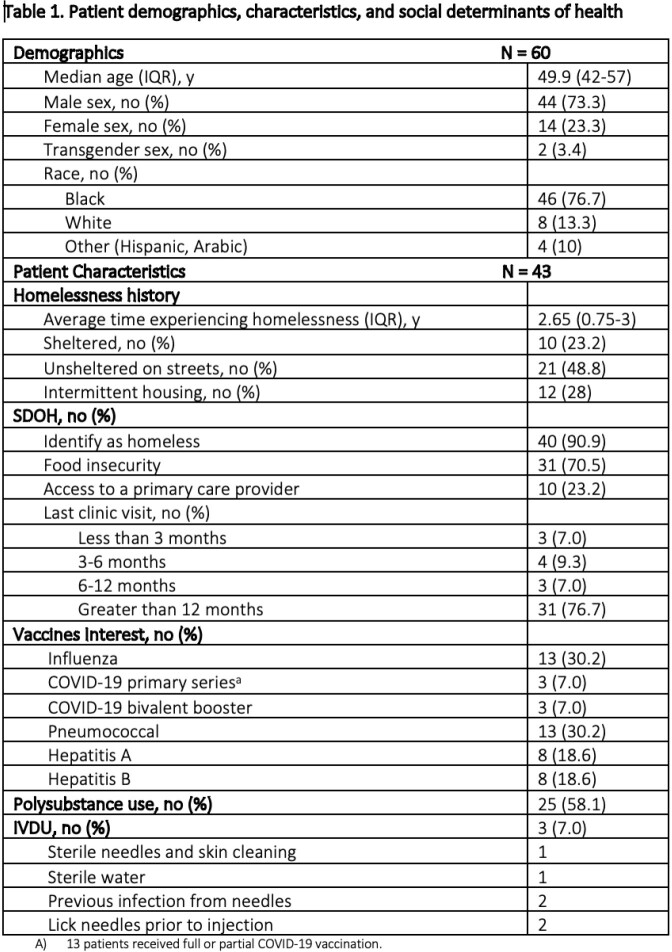

**Conclusion:**

We describe the implementation of a unique HCS with a targeted needs assessment for PEH in hospital. Challenges regarding the HCS included frequent reeducation of HCS members to provide recommendations and sign-out. One major patient barrier included lack of phone for follow-up. However, specific gaps for this population within ID expertise have been identified. HCS future goals are to focus on harm reduction education, STI prevention, and addressing vaccine hesitancy in PEH.

**Disclosures:**

**Marcus Zervos, MD**, Contrafect: Advisor/Consultant|GSK: Grant/Research Support|Johnson and Johnson: Grant/Research Support|Pfizer: Grant/Research Support

